# Coordination variety of phenyl­tetra­zolato and di­methyl­amido ligands in dimeric Ti, Zr, and Ta com­plexes

**DOI:** 10.1107/S2053229624007411

**Published:** 2024-08-23

**Authors:** Galina A. Bikzhanova, Ilia A. Guzei

**Affiliations:** a Department of Chemistry, University of Wisconsin-Madison, 1101 University Ave, Madison, WI 53706, USA; University of the Witwatersrand, South Africa

**Keywords:** solid angle, tetrazolato, phenyltetrazolato, η^2^-coordination, crystal structure, bridging

## Abstract

Three structurally diverse 5-phenyl­tetra­zolato (Tz) Ti, Zr, and Ta com­plexes, namely, (C_2_H_8_N)[Ti_2_(C_7_H_5_N_4_)_5_(C_2_H_6_N)_4_]·1.45C_6_H_6_ or (Me_2_NH_2_)[Ti_2_(NMe_2_)_4_(2,3-μ-Tz)_3_(2-η^1^-Tz)_2_]·1.45C_6_H_6_, (**1**·1.45C_6_H_6_), [Zr_2_(C_7_H_5_N_4_)_6_(C_2_H_6_N)_2_(C_2_H_7_N)_2_]·1.12C_6_H_6_·0.382CH_2_Cl_2_ or [Zr_2_(Me_2_NH)_2_(NMe_2_)_2_(2,3-μ-Tz)_3_(2-η^1^-Tz)_2_(1,2-η^2^-Tz)]·1.12C_6_H_6_·0.38CH_2_Cl_2_ (**2**·1.12C_6_H_6_·0.38CH_2_Cl_2_), and (C_2_H_8_N)_2_[Ta_2_(C_7_H_5_N_4_)_8_(C_2_H_6_N)_2_O]·0.25C_7_H_8_ or (Me_2_NH_2_)_2_[Ta_2_(NMe_2_)_2_(2,3-μ-Tz)_2_(2-η^1^-Tz)_6_O]·0.25C_7_H_8_ (**3**·0.25C_7_H_8_), where TzH is 5-phenyl-1*H*-tetra­zole, have been synthesized and structurally characterized. All three com­plexes are dinuclear; the Ti center in **1** is six-coordinate, whereas the Zr and Ta atoms in **2** and **3** are seven-coordinate. The coordination environments of the Ti centers in **1** are similar, and so are the ligations of the Ta centers in **3**. In contrast, the two Zr centers in **2** bear a different number of ligands, one of which is a bidentate η^2^-5-phenyl­tetra­zolato ligand that has not been observed previously for *d*-block elements. The di­methyl­amido ligand, present in the starting materials, remained un­changed, or was converted to di­methyl­amine and di­methyl­ammonium during the synthesis. Di­methyl­amine coordinates as a neutral ligand, whereas di­methyl­ammonium is retained as a hy­dro­gen-bonded entity bridging Tz ligands.

## Abstract 2

The first example of a nonbridging η^2^-coordination of a tetra­zolato ligand to a *d*-block element (Zr) is reported. Three related dinuclear 5-phenyl­tetra­zolato com­plexes of Ti, Zr, and Ta are structurally characterized.

## Introduction

Since the discovery of a facile synthesis of stable com­plexes of *d*-block elements with η^2^-coordinated pyrazoles (Guzei *et al.*, 1997 ▸), we have been inter­ested in the preparation of transition-metal com­plexes with similarly coordinated 5-substituted tetra­zoles. Tetra­zolato (tz) ligands, with their four N-atom lone pairs, display a wide variety of coordination modes (Massi *et al.*, 2018 ▸; Aromí *et al.*, 2011 ▸) from monodentate to polydentate, engaging multiple metal centers in coordination clusters or polymers. We were inter­ested in isolating discrete com­plexes of Ti, Zr, and Ta with a tz ligand that would display either 1,2-η^2^ or 2,3-η^2^ coordination, because such coordination by non­bridging tz ligands has not been reported for the *d*-block elements. The 1,2-η^2^ coordination by a nonbridging tz ligand has been reported for Ba (Kobrsi *et al.*, 2005 ▸), Cs (Hernández-Arganis *et al.*, 2018 ▸), K (Kobrsi *et al.*, 2006 ▸; Zheng *et al.*, 2003 ▸; Hu *et al.*, 2005 ▸), Sm (Evans *et al.*, 1988 ▸), and actinides U and Th (Browne *et al.*, 2016 ▸), whereas the 2,3-η^2^ coordination by a nonbridging tz ligand has only been reported for Ba (Kobrsi *et al.*, 2005 ▸).

**Figure 1:**
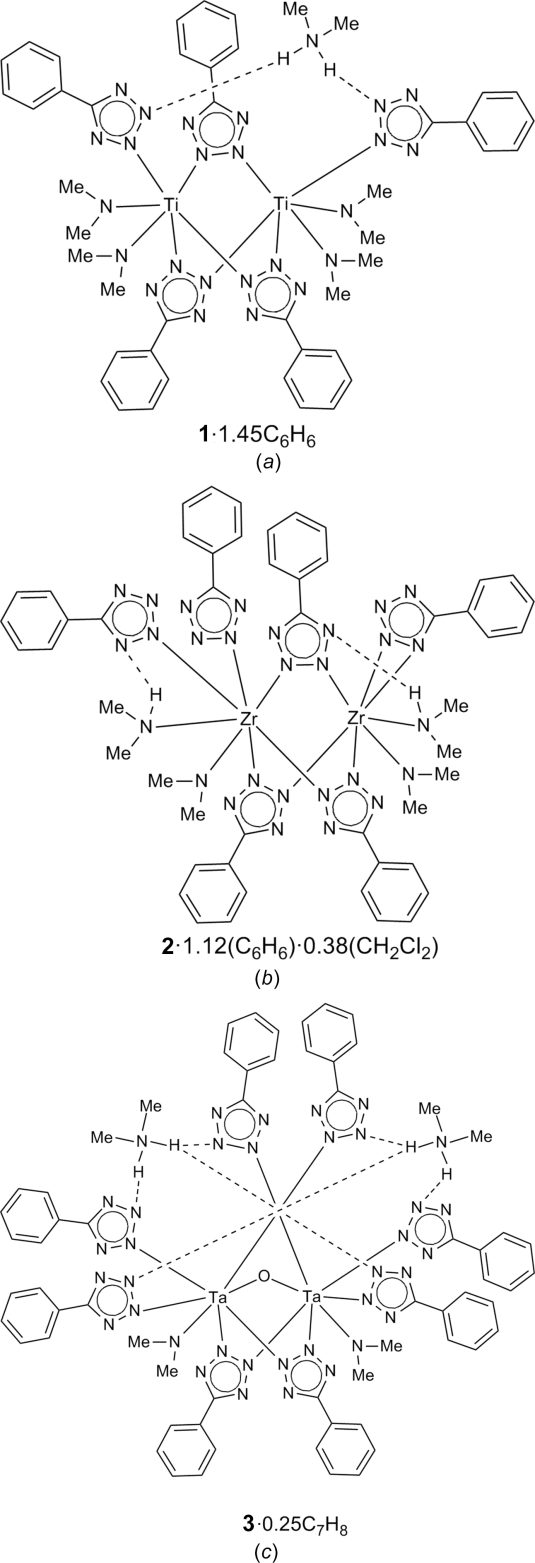
The Ti, Zr, and Ta com­plexes (*a*) **1**, (*b*) **2**, and (*c*) **3**, shown with their formulae.

The multidentate nature of the 5-phenyl­tetra­zolato (Tz) ligand selected for these studies promotes dimerization; thus, all three com­plexes reported herein are dimeric, and only the Zr com­plex contains a desired 1,2-η^2^ coordination (Fig. 1 ▸). To the best of our knowledge, this is the first structural report of a *d*-block element with a 1,2-η^2^ coordination of a nonbridging tz ligand. In all three com­plexes, Tz moieties are involved in hy­dro­gen-bonding inter­actions. Inter­estingly, the fate of the di­methyl­amido ligands present in the starting materials Ti(NMe_2_)_4_, Zr(NMe_2_)_4_, and Ta(NMe_2_)_5_ is different in the three synthetic reactions, as they are either retained or converted to either di­methyl­amine or di­methyl­ammonium. In the cases of Ti and Ta, the final products contain amido ligands and di­methyl­ammonium cations; in the Zr case, some groups remain amido, whereas some become coordinated di­methyl­amines. The di­methyl­amine is coordinated as a neutral ligand and di­methyl­ammonium is retained as a hy­dro­gen-bonded entity bridging Tz ligands. Numerous metal com­plexes with [Me_2_NH_*x*_]^*n*^, where *x* = 0, 1, or 2, and *n* = −1, 0, or +1, respectively, have been reported in the Cambridge Structural Database (CSD; Groom *et al.*, 2016 ▸).

Herein we report three related, but diverse, crystal structures of dinuclear com­plexes, namely, di­methyl­ammonium tris­(μ-5-phenyl­tetra­zolato-κ^2^*N*^2^:*N*^3^)bis­[bis­(di­methyl­amido)(5-phenyl­tetra­zolato-κ*N*^2^)titanium(IV)] benzene 1.45-solvate, (Me_2_NH_2_)[Ti_2_(NMe_2_)_4_(2,3-μ-Tz)_3_(2-η^1^-Tz)_2_]·1.45C_6_H_6_, (**1**·1.45C_6_H_6_), bis­(di­methyl­amido)-1κ*N*,2κ*N*-bis­(di­methyl­amine)-1κ*N*,2κ*N*-tris­(μ-5-phenyl­tetra­zolato-1:2κ^2^*N*^2^:*N*^3^)tris­(5-phenyl­tetra­zolato)-1κ^2^*N*^2^;2κ^2^*N*^2^,*N*^3^-dizirconium(IV)]–benzene–di­chloro­methane (1/1.12/0.38), [Zr_2_(Me_2_NH)_2_(NMe_2_)_2_(2,3-μ-Tz)_3_(2-η^1^-Tz)_2_(1,2-η^2^-Tz)]·1.12C_6_H_6_·0.38CH_2_Cl_2_ (**2**·1.12C_6_H_6_·0.38CH_2_Cl_2_), and bis­(di­methyl­ammonium) μ-oxido-bis­(μ-5-phenyl­tetra­zolato-κ^2^*N*^2^:*N*^3^)bis­[(di­methyl­amido)­tris­(5-phenyl­tetra­zolato-κ*N*^2^)tantalum(V)] toluene 0.25-solvate, (Me_2_NH_2_)_2_[Ta_2_(NMe_2_)_2_(2,3-μ-Tz)_2_(2-η^1^-Tz)_6_O]·0.25C_7_H_8_ (**3**·0.25C_7_H_8_). The Zr and Ta com­plexes crystallize with two symmetry-independent mol­ecules in the asymmetric unit; the second symmetry-independent mol­ecules are labelled in a similar fashion with ‘*A*’ as the label suffix.

## Experimental

### Synthesis and crystallization

Complex **1** was prepared and crystallized according to a published procedure (Guzei, 1997 ▸). Complexes **2** and **3** were prepared in a similar manner and crystallized from CH_2_Cl_2_/benzene and toluene, respectively. Adventitious oxygen became incorporated in Ta com­plex **3**; no further attempts were made to repeat its synthesis.

### Refinement

Crystal data, data collection and structure refinement details are summarized in Table 1 ▸. The diffraction data collections were performed by standard techniques on Bruker area-detector single-crystal diffractometers at 100 K with *APEX3* (Bruker, 2018 ▸), the data were scaled with *SADABS* (Krause *et al.*, 2015 ▸), and the structures solved with *SHELXS* and *SHELXT* (Sheldrick, 2015*a* ▸), and refined with *SHELXL* (Sheldrick, 2015*b* ▸) and *OLEX2* (Dolomanov *et al.*, 2009 ▸).

Compound **1**·1.45C_6_H_6_ crystallizes with a monoanionic dinuclear titanium com­plex, a di­methyl­ammonium cation, and ∼1.45 mol­ecules of benzene solvent in the asymmetric unit. The phenyl (Ph) rings at C12 and C26 are disordered over two positions, with major com­ponent contributions of 60.6 (13) and 77 (3)%, respectively. The Ph ring at C19 is disordered over three positions in a 43.4 (3):35.6 (3):21.0 (3) ratio. The disordered rings were refined with an idealized geometry and atomic displacement parameter restraints. The di­methyl­ammonium cation is disordered over two positions, with the major com­ponent present 54.3 (15)% of the time. The fully occupied solvent mol­ecule appeared to be benzene disordered over several positions. The other solvent mol­ecule was disordered over a crystallographic inversion center. A significant amount of time was invested in identifying and refining the disordered mol­ecules. Bond-length restraints were applied to model the mol­ecules but the resulting isotropic displacement coefficients suggested the mol­ecules were mobile. In addition, the refinement was com­putationally unstable. The SQUEEZE option (Spek, 2015 ▸) of *PLATON* (Spek, 2020 ▸) was used to correct the diffraction data for diffuse scattering effects and to identify the solvate mol­ecule. *PLATON* calculated the upper limit of volume that can be occupied by the solvent to be 910 Å^3^, or 15.5% of the unit-cell volume. The program calculated 246 electrons in the unit cell for the diffuse species. This approximately corresponds to one mol­ecule of benzene (42 e^−^) and 0.45 mol­ecules of either benzene or CH_2_Cl_2_ (19 e^−^) in the asymmetric unit. All derived results in the tables are based on the known contents. No data are given for the diffusely scattering species. This com­plex proved to be challenging to characterize and several data sets were collected on crystals isolated from several different syntheses.

Compound **2** crystallizes with two symmetry-independent dinuclear com­plexes in the asymmetric unit and several mol­ecules of crystallization solvents. The solvent mol­ecules occupy three different sites: (i) one fully occupied mol­ecule of benzene; (ii) a site shared by benzene and CH_2_Cl_2_ in a 64.3 (2):35.7 (2) ratio; (iii) a site shared by CH_2_Cl_2_/benzene­(orientation #1)/benzene­(orientation #2) in a 40.6 (2):35.7 (2):23.7 (2) ratio. The asymmetric unit content is **2**_2_.(benzene)_2.24_(CH_2_Cl_2_)_0.76_. The disordered benzene mol­ecules were refined with idealized geometries and atomic displacement parameter restraints. Atoms C19*S*–C24*S* of the 23.7%-occupied benzene mol­ecule were refined with identical isotropic atomic displacement parameters. The 40.6%-occupied di­chloro­methane mol­ecule was refined with geometrical restraints.

Compound **3** crystallizes with two symmetry-independent mol­ecules of the dinuclear dianioninc Ta com­plex, four di­methyl­ammonium cations, and half a mol­ecule of toluene solvent in the asymmetric unit. The toluene mol­ecule is disordered over a crystallographic inversion center and was refined with an idealized geometry (Guzei, 2014 ▸). The Ph ring at atom C52 is disordered over two positions, with the major com­ponent occupied 52 (2)% of the time. The disordered Ph rings were refined with an idealized geometry and atomic displacement parameter restraints.

## Results and discussion

The three com­plexes reported herein exemplify the remarkable structural diversity of dinuclear com­plexes of metals from periods IV, V, and VI with Tz and di­methyl­amido ligands (Table 2 ▸). These com­plexes possess different charges: the titanium com­plex is anionic, the tantalum com­plex dianionic, and the zirconium com­plex neutral. The charges are balanced by an appropriate number of di­methyl­ammonium cations that form N—H⋯N inter­actions with Tz ligands residing on different metal atoms. All metal centers bear di­methyl­amido ligands, but in addition the Zr centers bind to a neutral di­methyl­amine. Each metal is also ligated by two or three bridging μ-η^1^(N2):η^1^(N3) Tz ligands. The Ti, Zr, and Ta centers are further coordinated to terminal η^1^(N2) Tz ligands; inter­estingly, the titanium centers bear one such ligand, zirconium zero or two, and tantalum three. Strikingly, one Zr center does not bear terminal Tz ligands and instead features an η^2^(N1,N2) Tz moiety. The tantalum com­plex also contains a μ-oxo bridge, apparently due to a serendipitous incorporation of adventitious oxygen into the reaction mixture.

**Figure 2:**
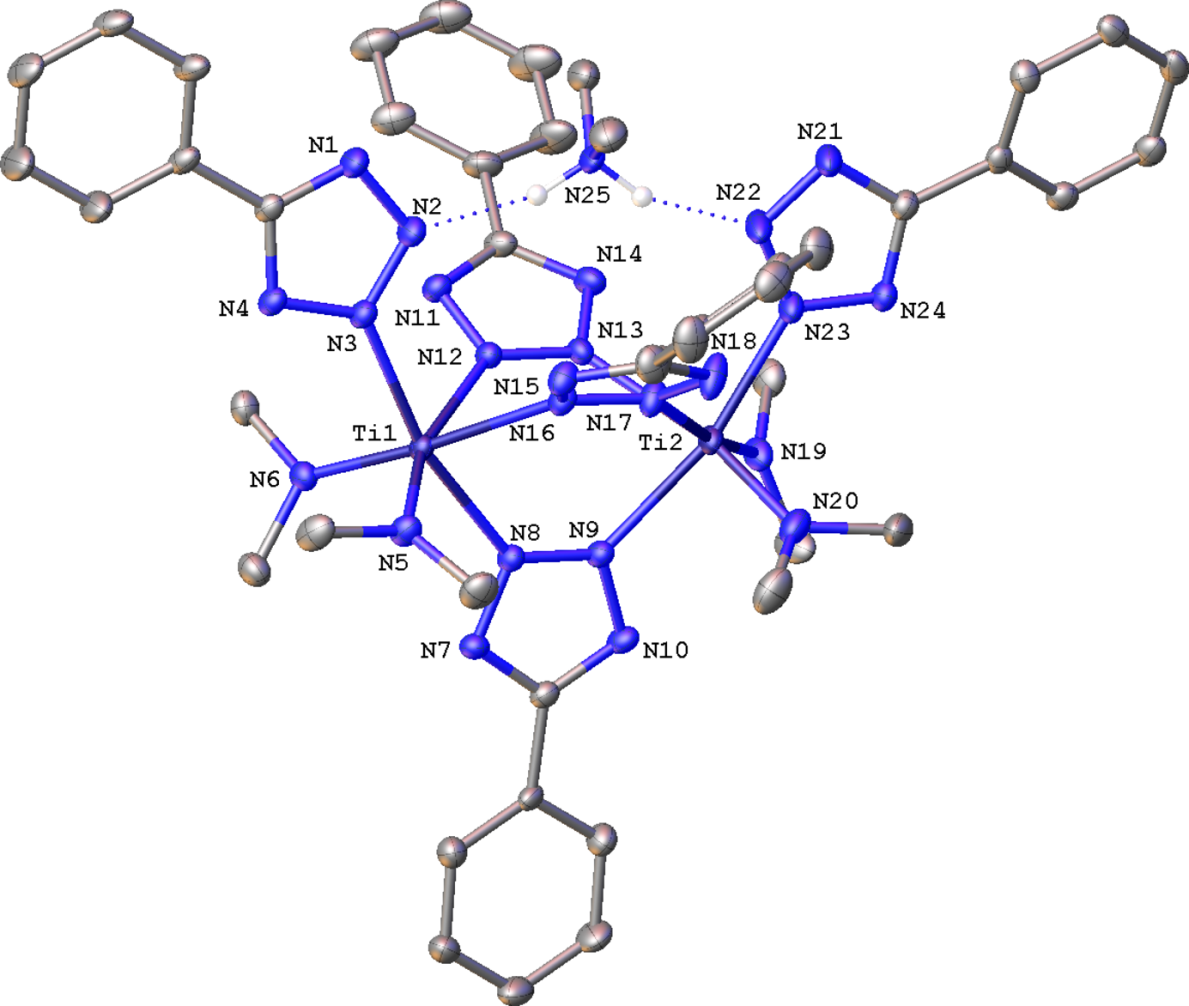
A mol­ecular drawing of **1**, shown with 30% probability displacement ellipsoids. Minor disorder com­ponents and H atoms not participating in hy­dro­gen bonds have been omitted.

In the monoanionic dinuclear com­plex **1**, the two six-coordinate Ti centers are separated by 4.2668 (6) Å (Fig. 2 ▸). Each Ti atom is ligated by two NMe_2_ ligands, three μ-η^1^(N2):η^1^(N3) Tz ligands, and one terminal η^1^(N2) Tz moiety. The charge is balanced by a di­methyl­ammonium cation that forms a hy­dro­gen bond to each terminal Tz ligand. The four Ti—NMe_2_ distances (Table 3 ▸) average 1.894 (5) Å and are in excellent agreement with the literature value of 1.91 (2) Å obtained by averaging 84 Ti—NMe_2_ bonds observed in 44 com­plexes reported in the CSD. The Ti—N(terminal Tz) bond lengths in **1** are very similar and expectedly longer at an average of 2.152 (2) Å. There is only one Ti com­plex with a monodentate η^1^(N2)-Tz ligand reported to the CSD, *i.e.* tris­(3,5-di-*tert*-butyl­pyrazolato)(5-phenyl­tetra­zolato)titanium(IV) (Yélamos *et al.*, 2001 ▸), and one with a neutral η^1^(N2)-TzH ligand, [TiCl_4_(PhCN_4_H)_2_] (Hill *et al.*, 2004 ▸). The Ti—N(Tz) and Ti—N(TzH) distances in these were determined to be 2.104 Å (low precision room-tem­per­a­ture data set), with an average of 2.272 (7) Å. Predictably, the Ti—N(bridging Tz) bonds in **1** are even longer, and fall into two groups because the com­position of the outer coordination sphere differs among the three bridging Tz ligands. Complex **1** may be described as pseudo-*C*_2*v*_-symmetrical, with one mirror plane roughly containing both terminal and one bridging Tz ligands. The Ti—N bond distances to the bridging N8-tetra­zolato ligand in the mirror plane are shorter [average 2.1970 (19) Å] than the other four Ti—N bridging distances [average 2.26 (2) Å], and the difference is statistically significant. This disparity can be explained by the relative location of these ligands. Atoms N8 and N9 of the bridging ligand in the mirror plane are *trans* to tz rings, whereas the ligated N atoms in the other bridging ligands are *trans* to π-electron-donating NMe_2_ units. The charge-assisted hy­dro­gen bonds between the [H_2_NMe_2_]^+^ cation and the Tz ligands are asymmetrical, with *D*⋯*A* and *D*—H⋯*A* parameters of 2.690 (8)/160 and 2.847 (8) Å/154° for one position of the disordered cation, and 2.742 (7)/156 and 2.864 (8) Å/169° for the other (Table 4 ▸).

**Figure 3:**
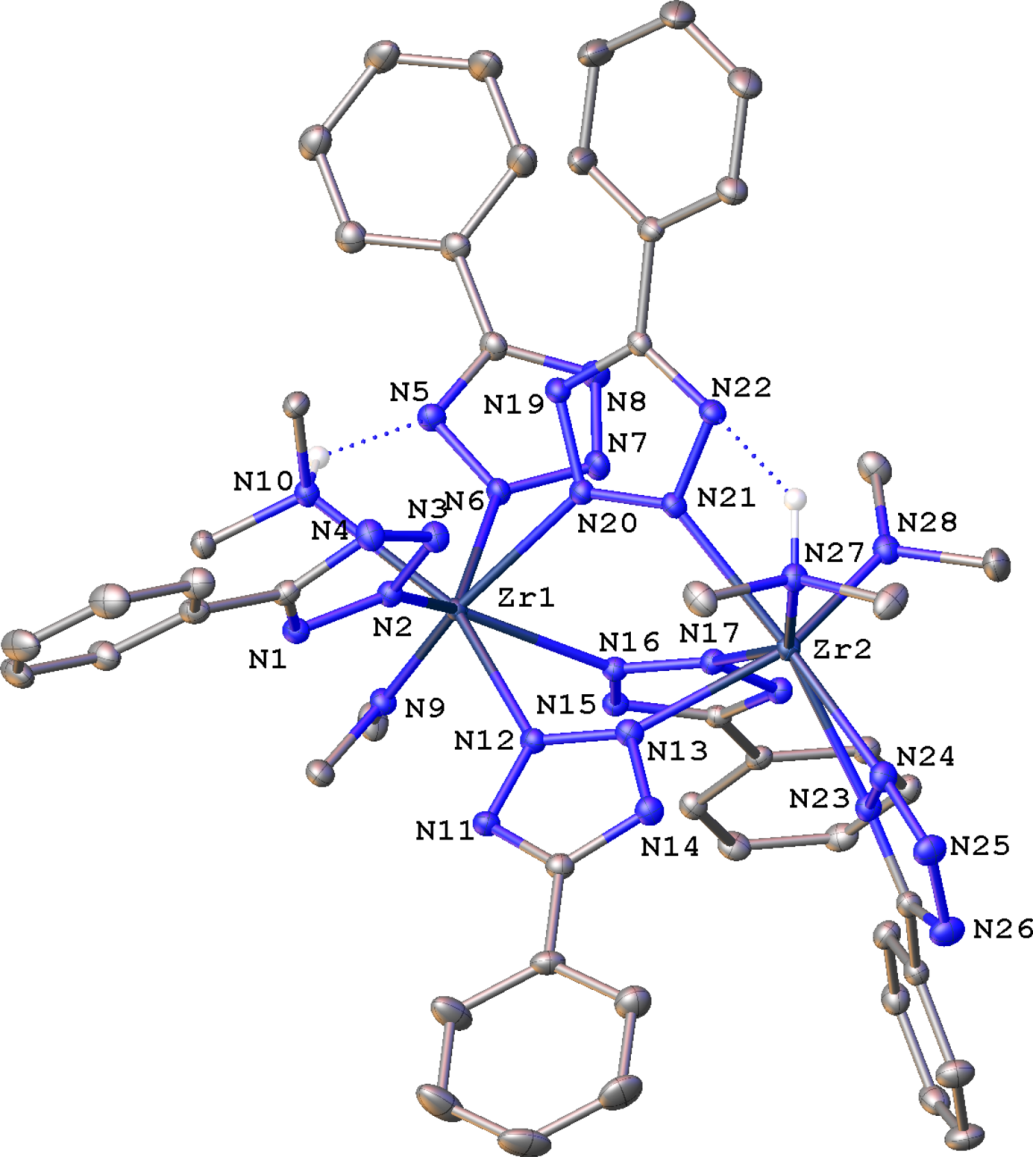
A mol­ecular drawing of **2a**, shown with 30% probability displacement ellipsoids. All H atoms not participating in hy­dro­gen bonds have been omitted.

**Figure 4:**
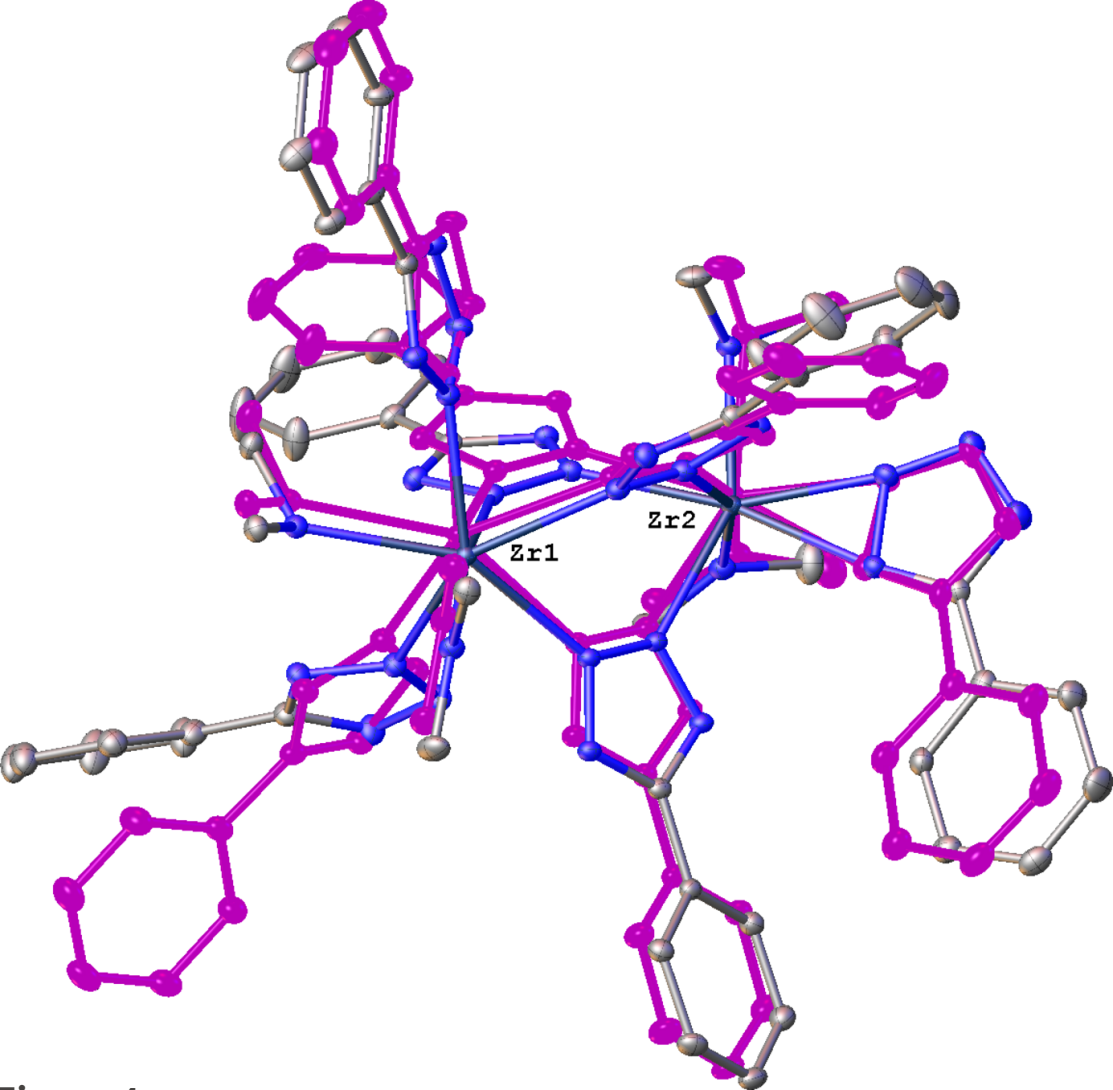
Superposition of **2a** and **2b**. All H atoms have been omitted. Complex **2b** is shown in monochrome.

The dinuclear Zr com­plex **2** (Fig. 3 ▸) crystallizes with two sym­metry-independent com­plexes designated hereafter **2a**, with atoms Zr1 and Zr2, and **2b**, with atoms Zr1*A* and Zr2*A* in the asymmetric unit. Complexes **2a** and **2b** are the only neutral ones in this series. They possess identical com­positions, but a superposition of the two mol­ecules clearly illustrates their conformational differences (Fig. 4 ▸). In the following discussion we will focus on Zr1 com­plex **2a** and provide data for **2b** as necessary. The two Zr atoms in **2a** are seven-coordinate, but have different coordination environments: Zr1 is ligated by a NMe_2_, HNMe_2_, three bridging μ-η^1^(N2):η^1^(N3) Tz ligands, and two terminal η^1^(N2) Tz ligands; Zr2 is ligated similarly, except that the two terminal Tz ligands are replaced by one bidentate η^2^(N1,N2) Tz fragment. The Zr⋯Zr separations in **2a** and **2b** are 4.5230 (3) and 4.5492 (3) Å, respectively, the latter being ∼0.27 Å longer than the Ti⋯Ti distance in **1**.

The η^2^(N1,N2) ligation mode of a nonbridging tz ring in Zr com­plex **2** is particularly exciting because it is the first structural report of a *d*-block element with this coordination mode. Based on a CSD search, such a coordination has been re­por­ted only for metals with atomic radii ≥ 1.75 Å (Slater, 1964 ▸), *i.e.* Ba with 5-(di­methyl­amino)tz (Kobrsi *et al.*, 2005 ▸), Cs with (1-phenyl-5-thione)tz (Hernández-Arganis *et al.*, 2018 ▸), K with 5-(pyrrolidin-1-yl)tz, 5-^t^Butz (Kobrsi *et al.*, 2006 ▸), Tz (Zheng *et al.*, 2003 ▸), and losartanide (Hu *et al.*, 2005 ▸), Sm with 1,5-penta­methyl­ene-tetra­zole (Evans *et al.*, 1988 ▸), and actinides U and Th with 5-Metz (Browne *et al.*, 2016 ▸). A bridging μ-η^1^(N3):η^2^(N1,N2) tz ring has been observed in dimeric com­plexes of Dy, Gd, Ho, Y, and Yb, and polydentate η^2^(N1,N2) tetra­zolato ligands have been reported for the alkali metals Na, K, Cs, and Rb. The only example with an η^2^(N2,N3) tz ligand has been reported for another large-atomic-radius-atom Ba (Kobrsi *et al.*, 2005 ▸). The atomic radius of Zr (1.55 Å) is noticeably smaller than the atomic radii of the elements listed above, but larger than those of Ti (1.40 Å) or Ta (1.45 Å), thus a large soft metal center is not a prerequisite for the formation of an η^2^-tetra­zolato com­plex.

In order to better understand the bidentate coordination mode of the tetra­zolato ligand, a series of density functional theory (DFT) minimizations were performed on a model com­plex Zr(Tz)Cl_3_ at the B3LYP/SDD level of theory. In these com­plexes, with largely ionic metal–ligand bonding, the com­plex with the 2,3-η^2^ ligand is 0.34 kcal mol^−1^ more stable than the 1,2-η^2^ analogue. Similar results were reported for model com­plexes tri­chloro­[(1,2-η)-5-methyl­tetra­zolato]titanium(IV) and tri­chloro­[(2,3-η)-5-methyl­tetra­zolato]titanium(IV), with the latter being more stable by less than 0.8 kcal mol^−1^ (Yélamos *et al.*, 2001 ▸). This is in slight contrast to the expectation based on the natural atomic charges com­puted for a free Tz^−^ anion: this analysis indicated that the N atoms in the 1- and 4-positions are more nucleophilic (by −0.21) than the 2- and 3-position N atoms, which means that a pair of adjacent atoms 1 and 2 (or 3 and 4) carries a larger combined negative charge than a combination of atoms 2 and 3, and that should favour the 1,2-η^2^ binding mode.

**Figure 5:**
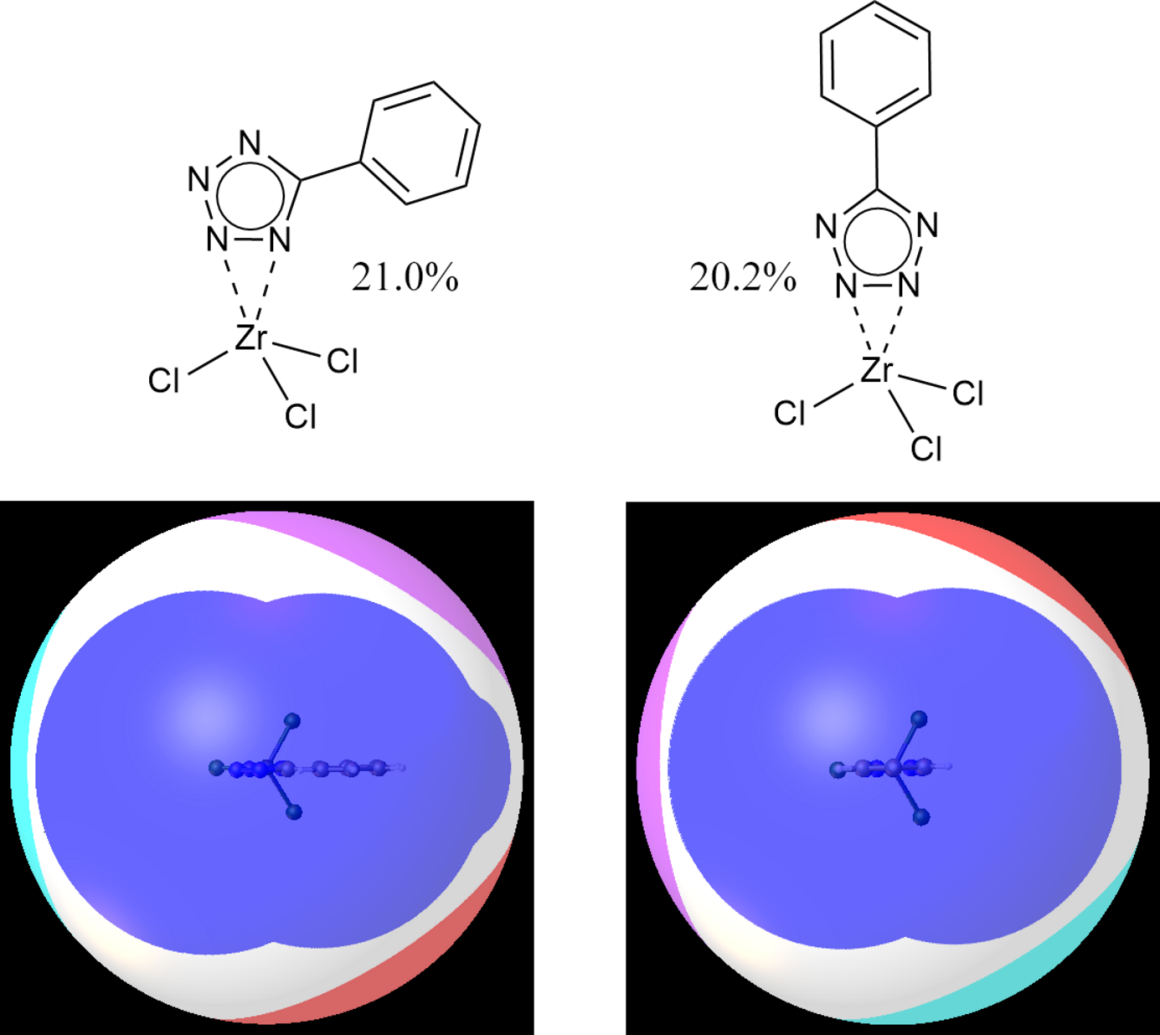
Model zirconium(IV) com­plexes used to evaluate the stability of the two coordination modes of the Tz ligand. The numbers are the *G*-parameters for the Tz ligands. The bottom figures illustrate how the bidentate ligands shield the metal – the colored portions of the sphere are ligands’ ‘shadows’ on a 12 Å sphere they would cast if the metal were replaced with a light source. The view is from the position of the Tz ligand down toward the metal. The shadow is blue for the Tz ligand, and red, fuchsia, and turquoise for the Cl atoms.

Steric considerations may play a decisive role when several ligand arrangements are similar in energy. The different coordination environments of the metal centers in **2** deserve a special scrutiny with the use of *G*-parameters that are based on ligand solid angles (Guzei & Wendt, 2006 ▸). The *G*-parameter describes ligand steric effects and represents the per­cen­tage of the metal coordination sphere shielded by a given ligand. In the model com­plex Zr(1,2-η^2^-Tz)Cl_3_ (Fig. 5 ▸), the bidentate ligand shields the metal to the extent of 21.0 (2)%, and in Zr(2,3-η^2^-Tz)Cl_3_, the corresponding parameter is 20.2 (2)%. The small difference between these values is not as counterintuitive as it may seem based on the position of the Ph ring, because the metal centers are primarily shielded by the ligated N atoms. The coordination environment of the metals in these model com­plexes is not crowded because the four ligands shield no more than 62% of the metal; thus, sterics is unlikely to explain the com­putational results. Either bidentate coordination mode of Tz is viable for zirconium and the rationale for the observed 1,2-η^2^ coordination may be found in the crystal packing forces.

Steric considerations become more com­plicated for experimentally observed geometries because the *G*-parameters may change depending on ligand conformations and orientations relative to the metal center. *G*-Parameters become smaller with increasing metal–ligand distances. Overall, atom Zr1 is shielded by seven ligands to an extent of 85% and atom Zr2 is shielded similarly up to 84% by six ligands. The *G*-parameters for the two terminal η^1^-Tz ligands on atom Zr1 add up to 22%, whereas the sole η^2^-Tz ligand on Zr2 shields 18% of the zirconium coordination sphere.

We note that the bond distances between the three bridging Tz ligands and Zr1 are slightly longer than those to Zr2; these ligands approach Zr2 closer and shield it by an additional 1% each. Consequently, the overall difference in the Zr1 and Zr2 *G*-parameters is only 1% (85 *versus* 84%).

Whereas the Zr—NMe_2_ distances to each metal center are similar (Table 5 ▸), the Zr—NHMe_2_ bond lengths are statistically significantly different. This distance to the seven-ligand-bearing Zr1 center is longer at 2.4073 (19) Å than to the six-ligand-ligated Zr2 atom at 2.3218 (19) Å. Both distances fall in the 2.308–2.574 Å range observed for Zr—NHMe_2_ distances com­puted for 50 such bonds in 37 com­plexes reported to the CSD. The HNMe_2_ ligands occupy different positions in the coordination spheres of each metal, as can be readily illustrated by the Zr⋯Zr—NHMe_2_ angles. The Zr1⋯Zr2—NHMe_2_ and Zr2⋯Zr1—NHMe_2_ angles span the range 107.65 (5)–145.45 (5)°. The di­methyl­amine ligands participate in intra­molecular hy­dro­gen-bonding inter­actions (Table 6 ▸). The HNMe_2_ ligand on the seven-ligand-bearing Zr1 center forms an N—H⋯N hy­dro­gen bond to a terminal Tz ligand, whereas the HNMe_2_ ligand on the six-ligand-ligated Zr2 atom forms a hy­dro­gen bond to a bridging tz ring.

The Zr—N(bridging) distances to Zr1 and Zr2 are dissimilar, and the difference is statistically significant. These bond distances to the seven-ligand-bearing Zr1 center [average 2.376 (7) Å] are longer than the respective bonds to Zr2 [average 2.314 (7) Å], a behaviour also observed for the di­methyl­amine ligands. Bond distances are expected to be longer for metals with higher coordination numbers, but, in this case, the formal coordination number is the same and the two metals differ only in the number of ligands.

The Zr1—N(terminal Tz) distances are expectedly longer than Zr2—N(bidentate Tz). The coordination mode of the η^2^(N1,N2)Tz may be described as ‘slipped’, with the Zr2—N23 distance to the N atom closest to the Ph ring being noticeably longer [2.3027 (19) Å] than the Zr2—N24 bond to the N atom in the second ring position [2.2238 (19) Å]. The difference can be rationalized in terms of the steric requirement of the Ph ring that does not allow a closer approach of N23 to Zr2.

**Figure 6:**
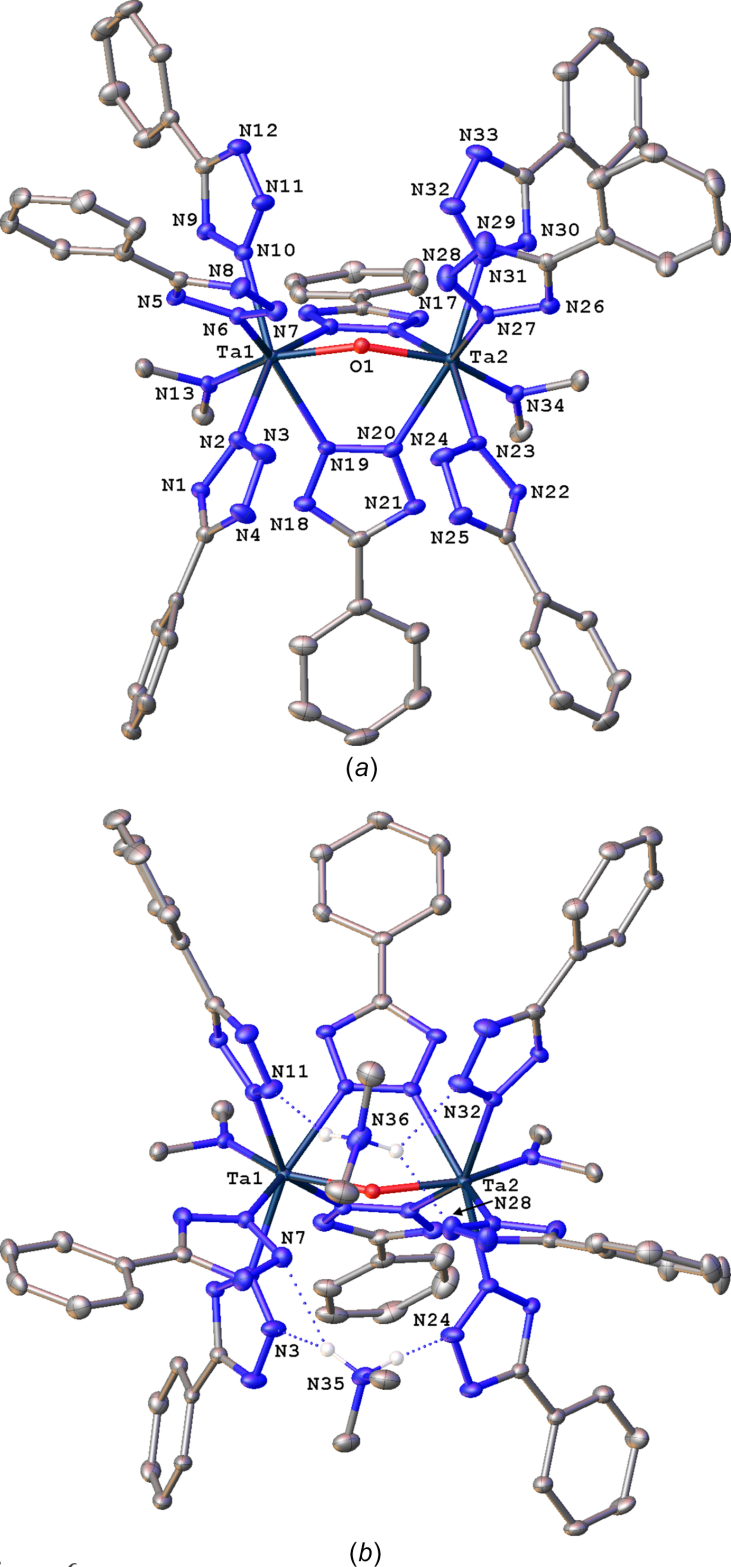
(*a*) A mol­ecular drawing of **3a**, shown with 40% probability displacement ellipsoids. All H atoms and minor disorder com­ponents have been omitted. (*b*) Hydrogen-bonding inter­actions between two H_2_NMe_2_ cations and dianionic Ta com­plex **3a**.

**Figure 7:**
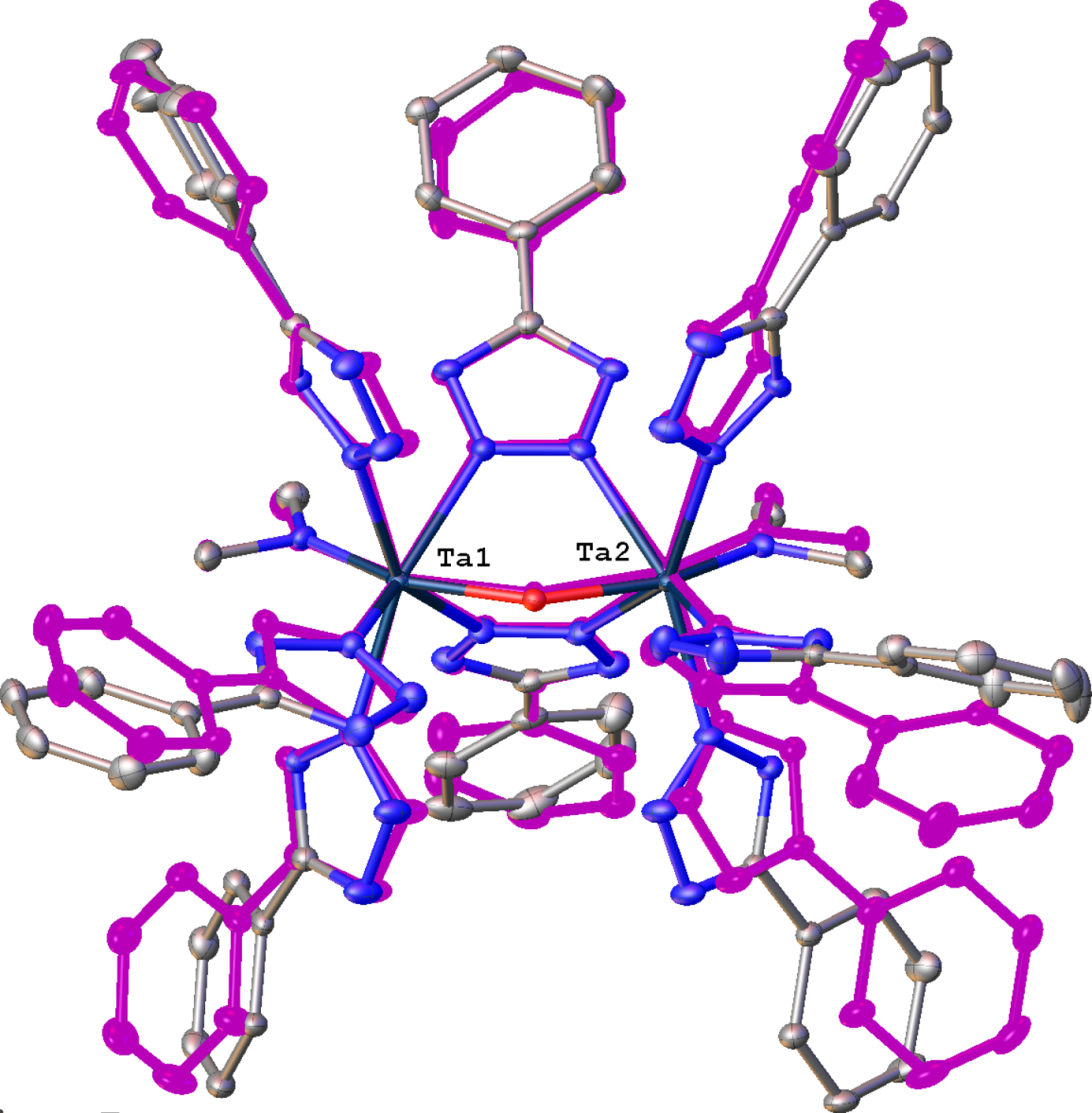
Superposition of **3a** and **3b**. All H atoms and minor disorder com­ponents have been omitted. Complex **3b** is shown in monochrome.

The structure of **3**·0.5C_7_H_8_ appears to be the first example of a structurally characterized Ta com­plex bearing a tz ligand. In the structure, there are two symmetry-independent dinuclear Ta com­plexes (**3a** and **3b**), with an identical com­position and very similar geometries about the metal centers (Fig. 6 ▸). The com­plexes differ mainly in the positions and conformations of the Tz ligands (Fig. 7 ▸). Each Ta center is seven-coordinated, with the coordination sphere com­prised of an NMe_2_ ligand, two bridging and three η^2^(N2)-terminal Tz ligands, and a bridging oxo ligand (Table 7 ▸). The two bridging Tz ligand rings connect the Ta centers in the same μ-η^1^(N2):η^1^(N3) arrangement observed for corresponding ligands in **1** and **2**. The Ta⋯Ta separation averages 3.645 (2) Å for the two com­plexes, and is the shortest metal–metal distance in this series by a large margin, undoubtedly due to the presence of the oxo bridge. The metal–ligand bond distances fall into three well-separated ranges: the Ta—NMe_2_ and Ta—O bond lengths are similar at ∼1.93 Å, the Ta—N(terminal Tz) are longer at ∼2.21 Å, and Ta—N(bridging Tz) are the longest at ∼2.27 Å. The com­plex exhibits a very approximate *C*_2*v*_ symmetry, with atoms Ta1, O1, and Ta2 defining one of the mirror planes. The dihedral angles between the tz-ring planes of the bridging ligands are 78.53 (10) and 75.43 (10)° in **3a** and **3b**, respectively, which is substanti­ally smaller than the ideal 120° angle that could be expected between three bridging ligands.

Two di­methyl­ammonium cations com­plete the second coordination sphere of each com­plex in a notable fashion by forming a total of six charge-assisted hy­dro­gen-bonding inter­actions. One [H_2_NMe_2_]^+^ forms one hy­dro­gen bond to a terminal Tz ligand on one Ta atom, and a bifurcated N—H⋯N(Tz) hy­dro­gen bond with its second acidic proton to two Tz ligands from the other Ta center (Table 8 ▸). These three hy­dro­gen-bonding inter­actions involve three of the six terminal Tz moieties. The other [H_2_NMe_2_]^+^ cation forms similar inter­actions with the three remaining Tz ligands, but the connectivity of the single and bifurcated hy­dro­gen bonds is reversed relative to the Ta centers. The single N—H⋯N hy­dro­gen bonds average 2.85 (2) Å, with the *D*—H⋯*A* angles in the range 149–161°. The bifurcated bonds are appreciably longer, with an average of 2.99 (5) Å, and their suboptimal *D*—H⋯*A* angles span the range 121–150°. Whereas there are no Ta–tz com­plexes and no closely related seven-coordinated Ta com­plexes with Ta—O—Ta bridges reported to the CSD, there are 164 com­plexes with a Ta—NMe_2_ fragment. In these, the Ta—N distances in the four-, five-, six-, seven-, eight-, and nine-coordinate com­plexes averaged 1.98 (3), 1.98 (3), 2.00 (5), 2.01 (3), 1.970 (15), and 1.990 (6) Å, respectively. The differences among these values are not statistically significant, and the expected trend of metal–ligand distance elongation with the increase in the metal coordination number is absent. The Ta—NMe_2_ distances in **3a** and **3b** are much shorter at 1.93 Å, and are among the shortest bond lengths of this type reported to the CSD. This is probably a result of the dianionic nature of the com­plexes.

## Conclusions

This work expands the number of structurally characterized com­plexes of Ti, Zr, and Ta with tetra­zolato ligands. All three com­plexes are dinuclear; the Ti center in **1** is six-coordinate, whereas the Zr and Ta atoms in **2** and **3** are seven-coordinate. The coordination environments of the Ti centers **1** and Ta centers in **3** are similar; however, the two Zr centers in **2** bear a different number of ligands, one of which is a bidentate 1,2-η^2^-Tz ligand, not previously observed for *d*-block elements. The starting materials for the chemical reactions leading to the isolation of **1**–**3** involved di­methyl­amido ligands that were (*a*) retained in the products, (*b*) converted to di­methyl­amine eliminated from the reaction mixture by gas evolution, (*c*) converted to di­methyl­amine and retained as a neutral ligand, and (*d*) converted to di­methyl­ammonium and retained as a hy­dro­gen-bonded entity bridging Tz ligands. These relatively large metal com­plexes crystallize with solvent-accessible voids in their lattices that are indeed occupied by ordered and disordered solvent mol­ecules. The refinement of the disordered solvent mol­ecules pre­sent­ed certain challenges but was accom­plished by standard techniques.

## Supplementary Material

Crystal structure: contains datablock(s) 1, 2, 3, global. DOI: 10.1107/S2053229624007411/ef3059sup1.cif

Structure factors: contains datablock(s) 1. DOI: 10.1107/S2053229624007411/ef30591sup2.hkl

Structure factors: contains datablock(s) 2. DOI: 10.1107/S2053229624007411/ef30592sup3.hkl

Structure factors: contains datablock(s) 3. DOI: 10.1107/S2053229624007411/ef30593sup4.hkl

CCDC references: 2373651, 2373650, 2373649

## Figures and Tables

**Table 1:** Experimental details Experiments were carried out with Mo *K*α radiation using a Bruker SMART APEX CCD area-detector diffractometer. Absorption was corrected for by multi-scan methods (*SADABS*; Krause *et al.*, 2015 ▸).

	**1**	**2**	**3**
Crystal data			
Chemical formula	(C_2_H_8_N)[Ti_2_(C_7_H_5_N_4_)_5_(C_2_H_6_N)_4_]·1.45C_6_H_6_	[Zr_2_(C_7_H_5_N_4_)_6_(C_2_H_6_N)_2_(C_2_H_7_N)_2_]·1.118C_6_H_6_·0.382CH_2_Cl_2_	(C_2_H_8_N)_2_[Ta_2_(C_7_H_5_N_4_)_8_(C_2_H_6_N)_2_O]·0.25C_7_H_8_
*M*_r_	1043.95	1351.41	1742.47
Crystal system, space group	Monoclinic, *P*2_1_/*c*	Triclinic, *P* 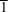	Monoclinic, *P*2_1_/*n*
Temperature (K)	100	100	105
*a*, *b*, *c* (Å)	11.6292 (9), 22.6611 (17), 22.3631 (17)	13.9631 (5), 16.7619 (6), 27.3054 (9)	22.5131 (8), 25.5101 (9), 26.7165 (9)
α, β, γ (°)	90, 93.857 (1), 90	86.902 (1), 81.154 (1), 89.969 (1)	90, 101.3830 (4), 90
*V* (Å^3^)	5880.0 (8)	6305.3 (4)	15041.8 (9)
*Z*	4	4	8
μ (mm^−1^)	0.32	0.43	2.98
Crystal size (mm)	0.4 × 0.3 × 0.2	0.4 × 0.3 × 0.2	0.39 × 0.32 × 0.24

Data collection			
*T*_min_, *T*_max_	0.576, 0.745	0.578, 0.745	0.4, 0.5
No. of measured, independent and observed [*I* > 2σ(*I*)] reflections	46383, 11558, 8907	70012, 25682, 20488	173076, 29545, 25534
*R*_int_	0.042	0.040	0.030
(sin θ/λ)_max_ (Å^−1^)	0.617	0.626	0.617

Refinement			
*R*[*F*^2^ > 2σ(*F*^2^)], *wR*(*F*^2^), *S*	0.050, 0.142, 1.09	0.039, 0.103, 1.01	0.027, 0.068, 1.09
No. of reflections	11558	25682	29545
No. of parameters	826	1671	1915
No. of restraints	898	67	79
H-atom treatment	H-atom parameters constrained	H atoms treated by a mixture of independent and constrained refinement	H-atom parameters constrained
Δρ_max_, Δρ_min_ (e Å^−3^)	0.53, −0.42	0.78, −0.98	2.48, −1.24

Computer programs: *APEX3* (Bruker, 2018 ▸), *SAINT* (Bruker, 2018 ▸), *SHELXS* (Sheldrick, 2008 ▸), *olex2.solve* (Bourhis *et al.*, 2015 ▸), *SHELXT* (Sheldrick, 2015*a* ▸), *SHELXL2018* (Sheldrick, 2015*b* ▸), *SHELXL* (Sheldrick, 2008 ▸), and *OLEX2* (Dolomanov *et al.*, 2009 ▸).

**Table 2:** Ligand com­position of com­plexes **1**–**3**

	Compound			
	**1**	**2**		**3**
Charge	−1	0	0	−2
	
Ligand	Metal center			
	Ti	Zr1	Zr2	Ta
NMe_2_	2	1	1	1
HNMe_2_	0	1	1	0
μ-Tz	3	3	3	2
η^1^-Tz terminal	1	2	0	3
η^2^-Tz terminal	0	0	1	0
μ-oxo	0	0	0	1
Number of hy­dro­gen-bonded Me_2_NH_2_^+^	1	0	0	2

**Table 3:** Selected bond lengths (Å) for **1**

Ti1—N3	2.1534 (19)	Ti2—N9	2.1970 (19)
Ti1—N5	1.892 (2)	Ti2—N13	2.2404 (19)
Ti1—N6	1.893 (2)	Ti2—N17	2.266 (2)
Ti1—N8	2.1967 (18)	Ti2—N19	1.901 (2)
Ti1—N12	2.2592 (19)	Ti2—N20	1.890 (2)
Ti1—N16	2.294 (2)	Ti2—N23	2.151 (2)

**Table 4:** Hydrogen-bond geometry (Å, °) for **1**

*D*—H⋯*A*	*D*—H	H⋯*A*	*D*⋯*A*	*D*—H⋯*A*
N25—H25*C*⋯N22	0.91	1.89	2.742 (7)	156
N25—H25*D*⋯N2	0.91	1.97	2.864 (8)	169
N25*A*—H25*E*⋯N22	0.91	2.00	2.847 (8)	154
N25*A*—H25*F*⋯N2	0.91	1.82	2.690 (8)	160

**Table 5:** Selected bond lengths (Å) for **2**

Zr1—N2	2.2935 (18)	Zr1*A*—N2*A*	2.2856 (19)
Zr1—N6	2.3340 (19)	Zr1*A*—N6*A*	2.3073 (19)
Zr1—N9	1.9798 (19)	Zr1*A*—N9*A*	1.987 (2)
Zr1—N10	2.4073 (19)	Zr1*A*—N10*A*	2.4095 (19)
Zr1—N12	2.3829 (18)	Zr1*A*—N12*A*	2.3907 (18)
Zr1—N16	2.3741 (18)	Zr1*A*—N16*A*	2.3651 (19)
Zr1—N20	2.3699 (19)	Zr1*A*—N20*A*	2.3822 (19)
Zr2—N13	2.3115 (19)	Zr2*A*—N13*A*	2.2969 (18)
Zr2—N17	2.3085 (17)	Zr2*A*—N17*A*	2.336 (2)
Zr2—N21	2.3227 (18)	Zr2*A*—N21*A*	2.3166 (19)
Zr2—N23	2.3027 (19)	Zr2*A*—N23*A*	2.290 (2)
Zr2—N24	2.2238 (19)	Zr2*A*—N24*A*	2.214 (2)
Zr2—N27	2.3218 (19)	Zr2*A*—N27*A*	2.3145 (19)
Zr2—N28	1.975 (2)	Zr2*A*—N28*A*	1.989 (2)

**Table 6:** Hydrogen-bond geometry (Å, °) for **2**

*D*—H⋯*A*	*D*—H	H⋯*A*	*D*⋯*A*	*D*—H⋯*A*
N10—H10⋯N5	0.85 (2)	1.96 (2)	2.732 (3)	151 (2)
N27—H27⋯N22	0.84 (2)	2.35 (2)	2.965 (3)	130 (2)
N10*A*—H10*B*⋯N5*A*	0.85 (2)	2.16 (2)	2.902 (3)	145 (2)
N27*A*—H27*A*⋯N22*A*	0.85 (2)	2.33 (2)	2.940 (3)	129 (2)

**Table 7:** Selected bond lengths (Å) for **3**

Ta1—O1	1.938 (2)	Ta1*A*—O1*A*	1.932 (2)
Ta1—N2	2.204 (3)	Ta1*A*—N2*A*	2.204 (3)
Ta1—N6	2.202 (3)	Ta1*A*—N6*A*	2.210 (3)
Ta1—N10	2.213 (3)	Ta1*A*—N10*A*	2.211 (3)
Ta1—N13	1.924 (3)	Ta1*A*—N13*A*	1.929 (3)
Ta1—N15	2.265 (3)	Ta1*A*—N19*A*	2.280 (3)
Ta1—N19	2.283 (3)	Ta1*A*—N15*A*	2.249 (3)
Ta2—O1	1.921 (2)	Ta2*A*—O1*A*	1.932 (2)
Ta2—N16	2.280 (3)	Ta2*A*—N20*A*	2.269 (3)
Ta2—N20	2.281 (3)	Ta2*A*—N16*A*	2.273 (3)
Ta2—N23	2.204 (3)	Ta2*A*—N23*A*	2.217 (3)
Ta2—N27	2.218 (3)	Ta2*A*—N27*A*	2.215 (3)
Ta2—N31	2.194 (3)	Ta2*A*—N31*A*	2.211 (3)
Ta2—N34	1.928 (3)	Ta2*A*—N34*A*	1.928 (3)

**Table 8:** Hydrogen-bond geometry (Å, °) for **3**

*D*—H⋯*A*	*D*—H	H⋯*A*	*D*⋯*A*	*D*—H⋯*A*
N35—H35*B*⋯N24	0.91	2.00	2.875 (4)	161
N35—H35*C*⋯N3	0.91	2.46	3.031 (4)	121
N35—H35*C*⋯N7	0.91	2.12	2.943 (4)	150
N36—H36*B*⋯N11	0.91	1.98	2.836 (4)	156
N36—H36*C*⋯N28	0.91	2.17	2.978 (5)	148
N36—H36*C*⋯N32	0.91	2.44	3.085 (4)	128
N35*A*—H35*D*⋯N3*A*	0.91	2.05	2.867 (4)	149
N35*A*—H35*E*⋯N24*A*	0.91	2.33	2.994 (4)	130
N35*A*—H35*E*⋯N28*A*	0.91	2.20	2.954 (4)	140
N36*A*—H36*D*⋯N7*A*	0.91	2.23	2.961 (4)	137
N36*A*—H36*D*⋯N11*A*	0.91	2.34	2.981 (4)	127
N36*A*—H36*E*⋯N32*A*	0.91	1.95	2.828 (4)	161
